# Fibrous Dysplasia in a 120,000+ Year Old Neandertal from Krapina, Croatia

**DOI:** 10.1371/journal.pone.0064539

**Published:** 2013-06-05

**Authors:** Janet Monge, Morrie Kricun, Jakov Radovčić, Davorka Radovčić, Alan Mann, David W. Frayer

**Affiliations:** 1 University of Pennsylvania Museum, University of Pennsylvania, Philadelphia, Pennsylvania, United States of America; 2 Department of Radiology, University of Pennsylvania Museum, University of Pennsylvania, Philadelphia, Pennsylvania, United States of America; 3 Croatian Natural History Museum, Zagreb, Croatia; 4 Department of Anthropology, Princeton University, Princeton, New Jersey, United States of America; 5 Department of Anthropology, University of Kansas, Lawrence, Kansas, United States of America; Max Planck Institute for Evolutionary Anthropology, Germany

## Abstract

We describe the first definitive case of a fibrous dysplastic neoplasm in a Neandertal rib (120.71) from the site of Krapina in present-day Croatia. The tumor predates other evidence for these kinds of tumor by well over 100,000 years. Tumors of any sort are a rare occurrence in recent archaeological periods or in living primates, but especially in the human fossil record. Several studies have surveyed bone diseases in past human populations and living primates and fibrous dysplasias occur in a low incidence. Within the class of bone tumors of the rib, fibrous dysplasia is present in living humans at a higher frequency than other bone tumors. The bony features leading to our diagnosis are described in detail. In living humans effects of the neoplasm present a broad spectrum of symptoms, from asymptomatic to debilitating. Given the incomplete nature of this rib and the lack of associated skeletal elements, we resist commenting on the health effects the tumor had on the individual. Yet, the occurrence of this neoplasm shows that at least one Neandertal suffered a common bone tumor found in modern humans.

## Introduction

Tumors are seldom documented in the human skeletal record in part because of their overall rarity. They are even more infrequent in the human fossil record, an effect of much younger age-at-death profiles and the fragmentary nature of most fossil remains. Here, we describe a Krapina Neandertal rib, which preserves bony indications of a fibrous dysplastic tumor.

The Krapina rock shelter is located on Hušnjakovo Hill, in the city of Krapina, some 55 km north of Zagreb, Croatia. Under the direction of Gorjanović-Kramberger, excavations at the site began in 1899 and continued until 1905, when almost 900 human bones were recovered, along with stone tools and thousands of faunal remains [1]–[4]. ESR and U-Series dating suggest a date between 120–130 kyr for the site with the entire stratigraphic sequence accumulating over a short 20 kyr time period or less [5]. The lithic collection has been characterized as Mousterian with side scrapers as the most common tool [6]. Krapina has yielded one of the largest samples of human skeletal remains accumulated from any Upper Pleistocene site. These human bones were recovered in a fragmentary condition, intermixed with the faunal and archeological sample. Only a few articulated bones have been found and there is no evidence of deliberate burial. Rather, cut marks and other evidence of bone processing suggest postmortem manipulation of the bones by other humans [7]. Among this sample of fragmentary cranial and postcranial elements, a number of pathologies have been identified, with most involving degenerative disease, blunt force trauma and dental hypoplasia [8]–[16].

## Discussion

**Figure 1:**
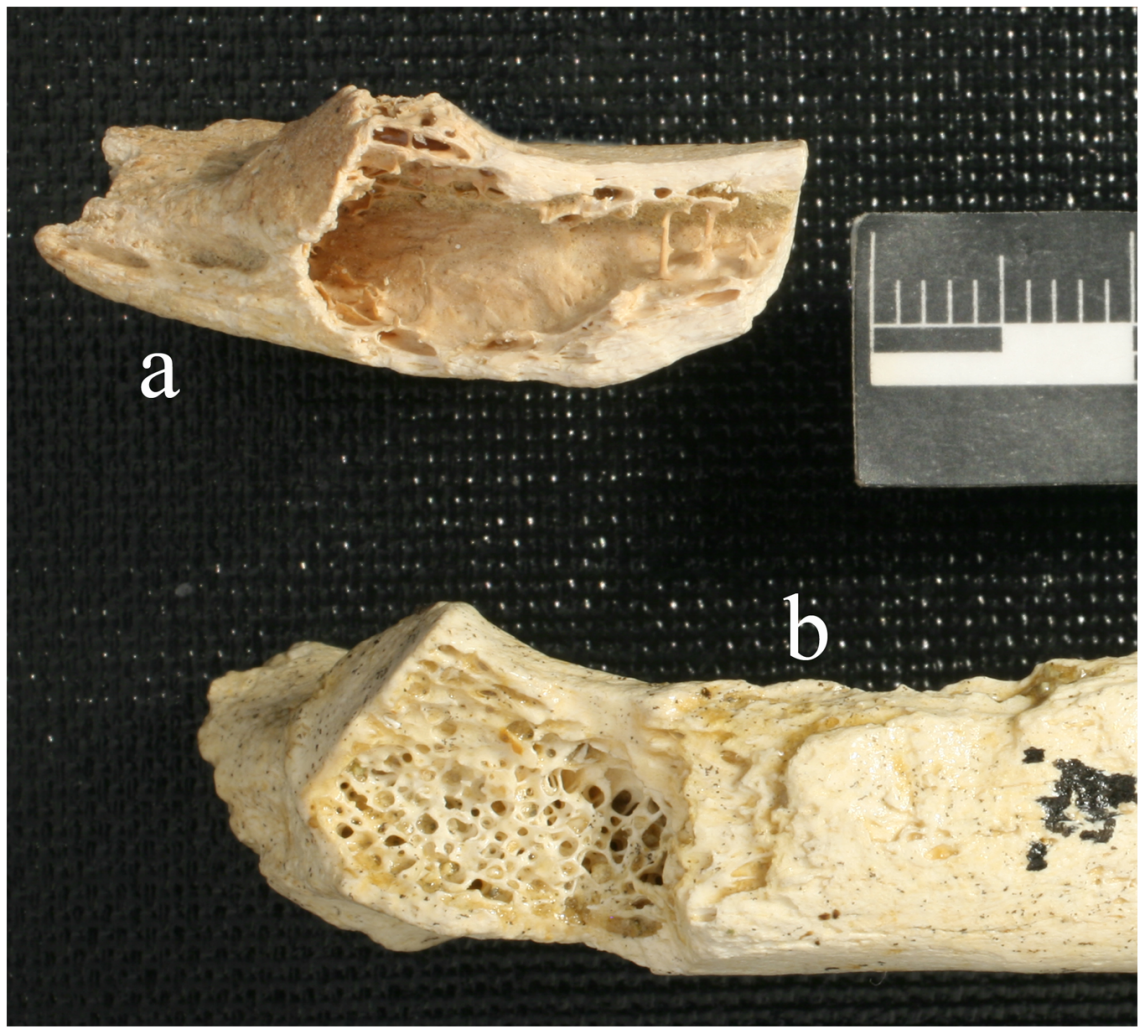
Krapina 120.71 in a caudal view (a). The large lesion is located above the tubercular facet and extends laterally. The trabeculae have been destroyed and the cortex appears expansive. The thin cortical bone forming the superior surface of the cavern was broken away postmortem. (b) Krapina 120.6 shows the normal pattern of bony trabeculae in the medullary space. The surface irregularities are post-mortem.

Krapina 120.71 is a 30mm long, left rib fragment containing about 2/3rds of the neck, most of the tubercular facet and a small section of the shaft. It appears to be a rib 3–6 [12] and matches up well with adult rib K120.1, a left 4–6 rib. It shares identical areas of preservation with 120.1 and both show deep muscular impressions on the neck. (See Table S1 for comparable ribs.) On K120.71 just lateral from the tubercular facet, the inferior surface shows a fresh break, exposing cortical bone and a large chamber (Fig. 1). External dimensions of the cavity are 18 mm in medial-lateral length and 7.6 mm at the maximum anterior-posterior breadth across the inferior, broken surface. The cavity’s internal aspect forms a smooth surface at the ceiling and on the anterior and posterior walls of the lesion. On the lateral-most portion of the bone where the rib narrows, two tiny bone spicules run across the medullary cavity in an anterior-posterior orientation. There are also some tiny spicules deep on the dorsal-most portion of the cavity, but for the most part the internal cavity is smooth and the cortical bone around it dense. Compared to Krapina 120.6, a left rib 3–5, where the cavity is packed with cancellous bone above the facet and continuing into the medullary fossa, the empty Krapina 120.71 medullary chamber is striking.

**Figure 2:**
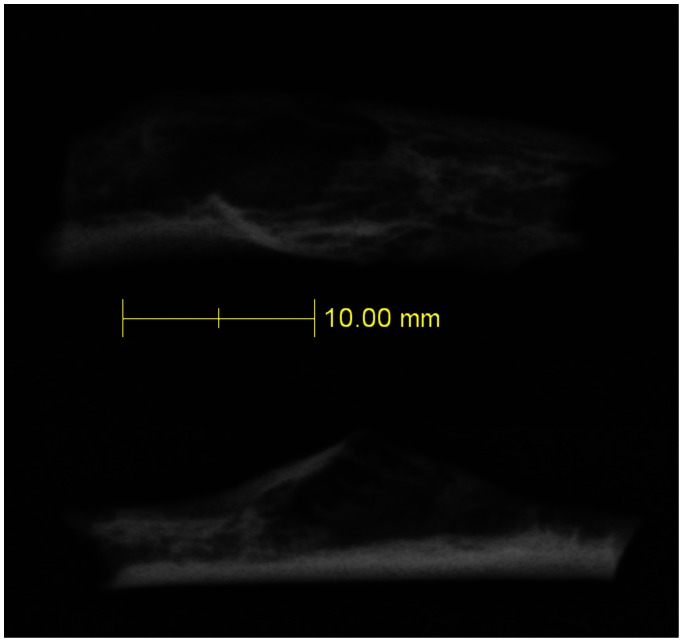
Radiograph of 120.71. Two views of Krapina 120.71 rib fragment: a: Superior/inferior radiograph view of Krapina 120.71 in position matching illustration in Figure 1; b: Lateral view in position matching the µ-CT scan series in Figure 3. The radiograph shows the full extent of the bony cavern excavated by the growth of the dysplastic mass. The lesion occupies most of the length of the fragment, but does not extend beyond the medial and distal borders. The full extent of the lesion is visible within this small rib fragment.

**Figure 3:**
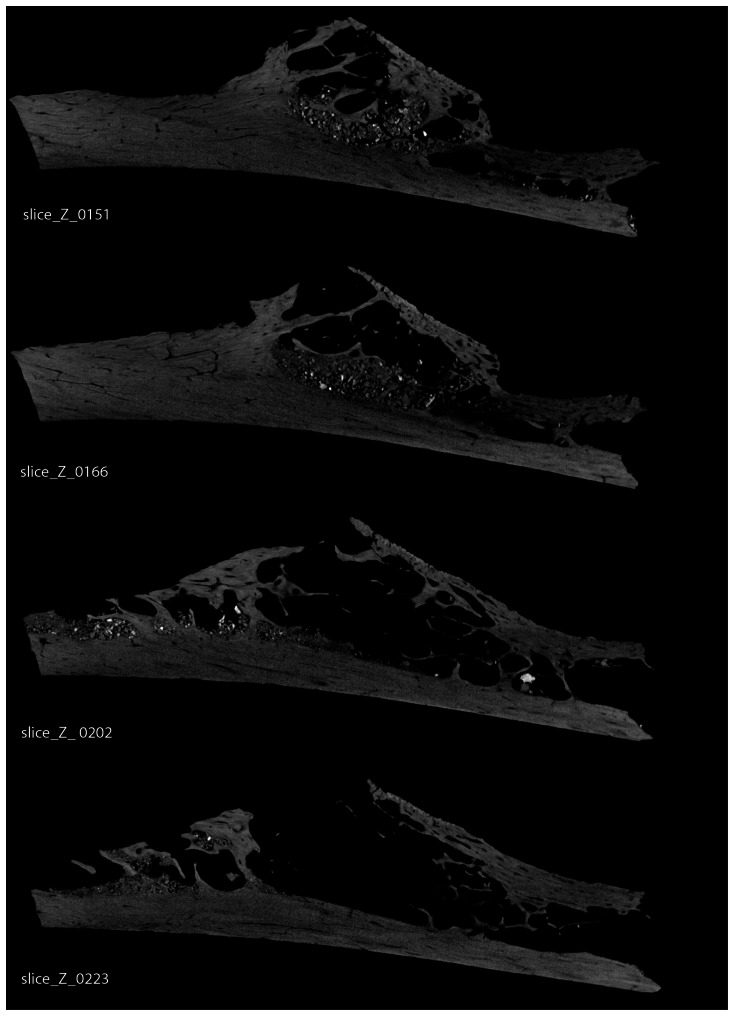
Krapina 120.71 was µ-CT scanned at 20 micron intervals (voxel size, 20 microns) across the long axis of the curved bone producing 495 individual images. Four slices are illustrated here showing the changing architectural detail of the internal tumor compartment. Loculations and vertical septations are clearly visible on each slice. Radiodense particles represent areas of adhering soil matrix that were part of the sedimentary environment.

A conventional radiograph of the rib shows an osteolytic lesion with a geographic pattern of bone destruction arising in the posterior-medial aspect of the shaft of the rib and showing a sharp non-sclerotic margin. The lesion is eccentrically expansive with a shell of periosteal bone (Fig. 2). From the µ-CT scans we calculated an internal maximum length of 24 mm, maximum breadth of 16 mm and a maximum depth of 10 mm. Using Analyze [R], contour outline measures of the internal void, taken every.5 mm from individual slice µ-CT scan projections, give a volume for the tumor of 78 mm^3^. Since the inferior most portion of the rib is broken away, the tumor must have been larger than this. The opposing cortex shows mild erosion, but no adjacent periosteal reaction. The original matrix forming the tumor disintegrated over time, and a µ-CT examination shows soil infiltration within the lesion. The µ-CT also shows a portion of the lesion that appears loculated with vertical septations. There has been erosion beneath the facet of the tubercle (Fig. 3).

The radiographic appearance of the lesion, the location of the lesion within the shaft of the rib and the estimated adult age of the individual suggest the most likely diagnosis is fibrous dysplasia. Fibrous dysplasia is a developmental disorder of bone in which lesions develop fibrous tissue and spicules of woven bone [17]. The medullary cancellous bone is replaced by fibrous tissue [18]. There may be one lesion (monostotic), several lesions, or multiple bone lesions (polyostotic). The monostotic form is the most common. The polyostotic form is often associated with skin pigmentation, may be associated with endocrine abnormalities and precocious puberty in females and rarely can have sarcomatous transformations. Rib lesions are often unilateral in the polyostotic form [19].

## Results

Fibrous dysplasia is the most common benign tumor/tumor–like lesion of ribs [20]–[21], accounting for 17% [22] to 33% [20] of primary rib lesions other than myeloma. In the monostotic form of fibrous dysplasia, 5.4% to 25% [17] of lesions occur in the ribs [17]–[18], [23]–[24]. In the polyostotic form of fibrous dysplasia, 55% of individuals have rib lesions [25] and the rib lesions are often unilateral [19]. Fibrous dysplasia occurs anywhere along the rib except at the ends [24].

The radiographic appearance of fibrous dysplasia of a rib is that of a slow-growing lesion with a geographic pattern of bone destruction that may appear round, elongated, or lobulated. It is centrally located in the marrow space, although it may appear eccentric as well [19], [24]. Bone “expansion” is common as the rib is a thin flat bone and the distortion may be symmetric or asymmetric. The margin of fibrous dysplasia is sharp and may be sclerotic or non-sclerotic. The matrix may appear radiolucent, may show peripheral trabeculae and appear loculated [22]–[23], may show mineralization with amorphous, faint, homogeneous increase in density (“ground glass”) appearance and on occasion may appear sclerotic [23]. The degree of radiographic density of the matrix depends upon the degree of spicules of woven bone within the fibrous matrix [17].

An enchondroma (chondroma) may have a similar radiographic appearance as the lesion presented, however, enchondromas develop at the sternal ends of ribs with 81.6% of rib enchondromas developing in the anterior aspect of the rib [22]. The matrix may show punctate, ring or arc-like calcifications indicative of cartilage matrix [24]. Enchondroma is the second most common benign rib lesion [22].

A post-traumatic lesion is unlikely as there is no evidence of a fracture and the expansive aspect of the lesion protrudes anteriorly. Any trauma to the rib would have occurred posterior. Chronic osteomyelitis is highly unlikely due to the lack of a sclerotic margin and the lack of periosteal reaction along the cortex opposing the expansive aspect of the lesion.

Chronic osteomyelitis is highly unlikely due to the lack of a sclerotic margin and the lack of periosteal reaction along the cortex opposing the expansive aspect of the lesion. Metastasis and myeloma are not likely since the relatively thick base of the periosteal shell suggests an underlying benign process [26]. In addition, it is unlikely that metastasis or myeloma existing in the medullary space, as seen in solitary hot spots (27) would have “expanded” the anterior cortex to such a degree without destroying or “expanding” the opposing cortex in this thin bone.

### Conclusions

Neoplastic bone disease, of both primary and secondary origin, is an exceptionally rare occurrence in the evolutionary fossil and archaeological record of human prehistory [28], until now extending back in time only 1,000–4,000 years [29]. Additionally, not only are primary bone neoplasms relatively rare in comparison to other neoplasms (accounting for a mere 7% of all soft and hard tissue forms of the disease [30]–[31]), but several other factors confound the study of bone disease in past populations. Osteolytic lesions on bone are difficult to classify without the accompanying soft tissue analysis (primarily, but not exclusively histologic) that is part of the medical process of the study of disease etiology. Compounding this are the processes associated with decomposition of the body after burial or deposition that serves to fragment, erode, distort, or completely destroy parts of the remaining bony tissues.

The frequency of neoplastic disease is strongly correlated with the relatively recent expansion of the human life span. This increases the frequency of neoplastic diseases, many of which are age-dependent [32]. Neandertals had average life spans that were at best half those modern populations, especially of people in developed countries [33]–[34]. Finally, it is recognized that environmental changes wrought by humans, compounded by population expansion, have resulted in an increase the types and the intensification of the pollutants within the environment, many of which are directly associated with neoplastic disease and were not part of environments in the past [35].

Given these factors, most argue that cases of neoplastic bone disease are rare in prehistoric human populations [27]–[28]. It is against this background that the identification of a 120,000+ year old Neandertal with a primary osteolytic lesion is surprising and one that provides insights into the nature and history of the association of humans to neoplastic disease.

## Supporting Information

Table S1 **A table of adult rib measurements for comparative Neandertal ribs from Krapina.**
(DOCX)
