# Supplementary material for: Fibrous Dysplasia in a 120,000+ Year Old Neandertal from Krapina, Croatia
Source: PLoS One. 2013 Jun 5;8(6):e64539. doi: 10.1371/journal.pone.0064539 (PMC3673952; doi:10.1371/journal.pone.0064539)
Supplement: Table S1 — A table of adult rib measurements for comparative Neandertal ribs from Krapina. (DOCX) [file pone.0064539.s001.docx]

Table legend: A table of adult rib measurements for comparative Neandertal ribs from Krapina.

Comparative rib measurements (in mm). All specimens are adults.

Identifications are from Radovčić & Wolpoff, et al (in prep)

Specimen Height of neck Superior breadth of neck Superior shaft height

dorsal to facet perpendicular to height ventral to facet

left

**120.71 (rib 3-6) 8.3 5.0 8.6**

120.1 (rib 3-4) 6.0 7.7 8.1

120.2 (rib 9-10) 6.7 6.9 8.7

120.3 (rib 9-10) 9.2 6.8 8.9

120.5 (rib 4-5) - - 7.8

120.6 (rib 3-5) 10.5 8.5 10.1

120.15 (rib 4-6) 9.5 4.8 9.1

120.17 (rib 3-5) - - 8.4

120.19 (rib 4-6) 5.7 8.9 9.0

120.24 (rib 4-5) 4.8 8.2^1^ 7.3

right

120.7 (rib 9-10?) 9.4 7.5 -

120.8 (rib 5-7) - - 7.7

120.12 (rib 6-8) - - 8.0

120.23 (rib 3-4) - - 9.0

^1^ – estimated

Radovčić J, Wolpoff MH et al (in prep) The Krapina hominids: An illustrated catalog of the skeletal collection. Zagreb: Mladost and the Croatian Natural History Museum.
